# Staying the Course? Challenges in Implementing Evidence-Based Programs in Community Mental Health Services

**DOI:** 10.3390/ijerph111010752

**Published:** 2014-10-16

**Authors:** Urban Markström

**Affiliations:** Department of Social Work, Umeå University, 901 87 Umeå, Sweden; E-Mail: urban.markstrom@socw.umu.se; Tel.: +4690 7865446

**Keywords:** evidence-based practice, community mental health, implementation, assertive community treatment, integrated care

## Abstract

This paper focuses on the second phase of the deinstitutionalisation of mental health care in which the development of community-based interventions are supposed to be implemented in local community mental health care systems. The challenge to sustainable implementation is illustrated by the Swedish case where the government put forward a national training program that sought to introduce Assertive Community Treatment (ACT) for people with severe mental illness. This study is based on document analysis and qualitative interviews with actors at the national, regional, and local levels covering a total of five regions and 15 municipalities that participated in the program. The analysis of the national experiences is put in relation to both research on public administration and policy analysis as well as to current research on implementation of evidence-based programs. The results showed a “drift” of the original model, which had already begun at the policy formulation stage and ended up in a large number of different local arrangements where only a few of the original components of ACT remained. We conclude that issues with implementation can only be fully understood by considering factors at different analytical levels.

## 1. Introduction

Over the past several decades the deinstitutionalisation of mental health services has taken place in Western countries, and this has led to radical changes in how mental health care is provided. However, there are still significant differences between countries and regions. Among European countries, there are still systems that rely heavily on hospital-based care and institutional arrangements [1]. To support the reform process, both the European Union and the World Health Organisation have launched action plans for community mental health services [2,3]. For countries that were early starters, it is possible to identify two stages in the deinstitutionalisation process. The first phase focused the downsizing of the number of in-patient beds at mental hospitals and the transfer of patients to community-based settings. The new system was composed of services like large group homes, and sheltered services for day care [4]. In the second phase, critiques have been put forward against these solutions. In Sweden, for example, researchers highlight the risk of replacing the mental hospitals with similar institutional arrangements and emphasize how this approach decreases the potential for community integration [5,6,7,8]. Other characteristics for the second phase are recovery-oriented approaches and person-centred care. These ideas are sometimes established at central policy levels [4,6,9]. An additional feature of the second phase is the emphasis on specific psychosocial interventions. Today there exist internationally evaluated methods for areas such as integrated care and support, family interventions, and vocational rehabilitation. The current level of evidence is investigated through meta-analysis and put together in national guidelines. Such documents are used to steer policy development [10,11], and to facilitate the implementation of the programs in everyday practice. Sometimes the guidelines are introduced by national associations of mental health professionals, but in the Swedish case the National Board of Health and Welfare (a state-run authority) have been responsible for the construction and dissemination of these guidelines [12,13]. The aim of this paper is to analyse and discuss the challenges of “translating” Evidence Based Practices (EBP) in order to implement them in local community mental health services. The point of departure is a study of a governmental initiative in Sweden where a large training program was launched in order to introduce Assertive Community Treatment (ACT) in all parts of the country.

### 1.1. The Implementation Dilemma

With a number of evidence-based methods available, the issue of implementation has been given more attention. How can the research findings best be transferred to local practice? The experiences of community mental health services are so far discouraging, especially concerning the translation of knowledge from an experimental environment to real life settings [4,14,15,16,17]. However, there are also experiences from large-scale implementation projects which demonstrate that successful implementation and high program fidelity can be reached through supports including consultant trainers and toolkits, in combination with an active local leadership [18,19]. Implementation research can be divided into two traditions: an established one that is anchored in research on public administration, and a field that focus specifically on implementation of EBP [20,21]. The former has a rich tradition based above all on the study of political reforms and policy implementation. Hill and Hupe have [22] pointed out some critical steps in the process of policy implementation. These illustrate the importance of including different levels in the analysis because the characteristics of the policy itself tend to affect the implementation process. For example, if the policy is vague or contradictory it will allow for different local interpretations that in turn can create a practice that diverges from the stated goals. However, if the policy is very detailed it can decrease the ability for necessary adjustments among local governments and in the long run can negatively effect the legitimacy of the central actors. The designs of national initiatives, which are developed by administrative bodies, often impact the behavior of both local service providers and other stakeholders that are involved in the current policy area. Furthermore, the vertical process that links the policy formation with its street-level implementation is of importance. Traditionally, this dynamic has been seen from a top-down perspective and in terms of governance strategies like legislation, economic policies, or by norm steering. A categorisation have been made in terms of “hard-governance”—where the implementation is directed by detailed regulations that limit discretion on the local level—and “soft-governance”—where a great deal of the decision-making power to is left to the service providers [23,24]. Another step described [22] is the response among the local agencies, which can be divided into the disposition of the involved agencies and that of the agency’s front-line staff. Here there is an extensive body of research, not the least of which is connected to the concept of “street-level bureaucracy” [25]. In addition, horizontal inter-organisational relationships between agencies affect the implementation of policy, especially in areas where services are supposed to be integrated with contributions from more than one service provider. Finally, the responses from the users, or those affected by the policy, can have a impact on the implementation. Strong support from, for example, client associations facilitate the establishment of welfare policies [20,22].

The understanding of implementation challenges can be deepen through research on EBP. Influential contributions to this field have been made by Fixen and colleagues [26,27], Greenhalg and colleagues [28], Damschroder and colleagues [29], and Durlak and DuPre [30]. These authors have all described components that influence the implementation and that affect the efficacy of current interventions. There is consensus about the importance of having an active approach in implementation work and that many of the components are compensatory. Careful recruitment of staff, on-going training and supervision, systems for feedback, and technical support are all components that have empirical support [26,31]. Current research suggests that things like verified local needs of the program, stable financing, a good fit between the program and the host setting, and access to experts are important for implementation [27,30,31]. Meyers and colleagues [31] stress that most of the important steps should be addressed before implementation begins and that good results are best achieved through a combination of activities that include assessment, negotiation, group planning, and critical analysis.

The research traditions described above have suffered from their different limitations, for example, the research on policy implementation has not made full use of data that can grasp local circumstances. The research on EBP implementation is often characterised by an absence of analysis concerning the system level. There is, as pointed out, for example, by Johansson [20] and Nilsen and colleagues [21], a need for analysis that attempts to combine features from the two positions. The present study tries to illustrate the interplay between components on different levels through experiences of both national and local actors.

### 1.2. An Introduction to the Swedish Case and Assertive Community Treatment (ACT)

The Swedish mental health field has changed rapidly over the last decades, and this has been to a large extent through government initiatives. The mental hospitals have been replaced by psychiatric wards at general hospitals and by different kinds of settings for residential services. Today, most of the support for people with psychiatric disabilities is divided between two major organisations. The first consists of the county councils that are responsible for in- and out-patient psychiatric care. The second consists of the social service agencies of the local municipalities that are responsible for all kind of community-based support such as housing and occupational support for the target group. Much emphasis from the national level is put on creating collaboration between psychiatric care and the social services. In 2011, the National Board of Health and Welfare launched national guidelines for psychosocial interventions for people with schizophrenia [12]. One of the few programs that reached the highest level of recommendation was ACT.

ACT is an integrated and comprehensive model for treatment and support that was developed in the US in the late 1970s. The aim was to facilitate a life in the community for people with severe mental illness who had extensive and complex needs [32]. ACT has undergone substantial modifications since the pilot-programmes, and today it is one of the most well-established models in community mental health. Despite the fact that the model has been implemented in different programs, there is relatively broad consensus concerning the core components. In particular, the support should be provided by a multi-professional team with the primary responsibility for providing person-centred care. The team should have a low caseload and deliver services within the community. Community resources are the primary partners in the work of the team, and the services that are offered should not have any time limits [33,34]. A meta-analysis of ACT shows that it has significant advantages with regard to treatment compliance and the use of in-patient care as well as for providing a more stable housing situation for the clients [35]. Internationally, the effectiveness of the program has since become the subject of debate, especially after studies in the UK failed to replicate the results from earlier studies in the US [36]. According another research review, the results of the studies on ACT are related to the total amount of in-patient care being provided to the clients at the time of baseline—the program seems to show the strongest effect among participants who are high consumers of hospital care [37]. Consequently, the Swedish national guidelines emphasise that ACT should be reserved for people with extensive needs and who have a history of low compliance and large amounts of hospital stays [12].

The ACT model includes a case manager function where most of the team members are responsible for coordinating support for the clients. This function is the core feature of a number of case management (CM) models that are designed with different intensities and with different caseloads. Available research states that to be effective the programs need to be comprehensive in the sense that the role of coordinator should be combined with teamwork where both clinical and social support is included [33]. In the Swedish context, one of the challenges for implementing intensive CM or ACT is to deliver integrated services in a welfare system where the responsibility is divided between two different authorities—the psychiatric care services and the social services.

## 2. Methods and Materials

The national initiative that serves as the illustrative case for this article was initially based on a governmental commission, “The National Coordination of Mental Health” [38]. The commission presented a proposal to the government’s social ministry regarding a large project for educational support. The government decided to disburse 2.2 million EUR to a project aiming to facilitate the development of “case managers that are working according to ACT”. A mandate was given to a Swedish university, which in turn made an agreement with a private company that specialised in community psychiatry. The education and training program that was designed in the project consisted of a five-week course for frontline staff or team members in psychiatry and social services and a 20-week training program for people who planned to work as case managers in the future. In addition, the education provider was responsible for offering local implementation support. The project as a whole covered 15 municipalities located in five regions in Sweden. To grasp experiences from both the planning and implementation phases, and to gather information from the local settings, data were collected at several levels.

To investigate the policy and the strategies behind the project, semi-structured telephone interviews were conducted with the following three key persons: the initiator of the project from the National Coordination of Psychiatry, the civil servant on the National Board of Health and Welfare who was responsible for monitoring the project, and the person at the university who was responsible for conducting the training programs. The interviews lasted for about one hour each and were recorded and transcribed. The interview guide covered questions about the background and the motives behind the initiatives, the ACT model, and the strategies for implementing and monitoring the training program. In addition, the proposal and the government’s decision were read and analysed.

To better understand the training programs, all material (including curricula, schemes, and working material) from both the short and the more extensive program were collected and analysed. Semi-structured telephone interviews were conducted with key persons in all five regions directly after the short training program had ended. These were often people with a specific commission to coordinate and plan the implementation of the training programmes in the regional context. They were organisationally based in either the psychiatric or social services, and their regular positions were either as front-line staff or first-line managers. Most often, these informants had additional responsibilities at the same time that they took part in the education. In total, 12 people were interviewed, six of whom worked in the social service organisation while the other six worked in psychiatric services. The interviews were recorded and transcribed verbatim and covered questions regarding both the structure and the content of the program. The situation after the training activities was studied through follow-up interviews with the same categories of informants as in the first set of interviews. These follow-up interviews were conducted about one year after the end of the short program and a few months after the end of the long training program for prospective case managers. Nine people were interviewed in the second round, and the interview guide mainly covered questions about the experiences of regional and local implementation of the program.

In order to deepen the understanding of the local experiences, four municipalities were chosen for analysis based on the principle of variation regarding size and geographical location. The group consisted of one large municipality in the north of Sweden, one medium-sized municipality in the south of the country, and two municipalities located in the western part of the country, one small and one medium-sized. Interviews were carried out in each municipality directly after the long training program was finished. The common procedure was to interview managers of psychiatric and social services and the staff that had participated in both the short and the longer training program. On average, about ten interviews were conducted at each site (see Table 1, Table 2, Table 3 and Table 4). The staffs were often interviewed in groups of two to five people. Besides questions about the experiences from the training programmes, the interviews also included questions about the strategies for local and sustainable implementation of the ACT model.

To assess the relation between the local practice and the original model of ACT, the plan was to use a well-established instrument for measuring program fidelity, the Dartmouth Assertive Community Treatment Scale (DACTS) [39]. Since it became increasingly clear that the instrument was not applicable for use with the current teams however, no results from the fidelity assessments are presented in this paper.

**Table 1:** Descriptions of the collected data. Interviews with actors at the national level.

National Coordination of Psychiatry	1
National Board of Health and Welfare	1
Project leader, Swedish university	1

**Table 2:** Description of the collected data. Interviews with key persons at the regional level.

Region (Number of Municipalities)	Representative from the Municipality	Representative from the Psychiatric Care Services	Others	Total Number of Interviews
North (3)	2	1	--	3
Middle I (4)	1	1	--	2
Middle II (3)	1	1	--	2
South (2)	2	1	--	3
West (3)	--	1	1	2

**Table 3:** Description of the collected data. Interviews at four sites.

Municipality	Managers	Other Functions	Frontline Staff	User Associations	Total Number of Interviews
West I	5	1	6	--	12
West II	3	1	4	--	8
North I	2	1	9	--	12
South I	2	2	4	3	11

**Table 4:** Description of the collected data. Interviews with key persons at the regional level during the follow-up.

Area	Representative from the Municipality	Representative from the Psychiatric Services	Others	Total Number of Interviews
North (3)	1	1	--	2
Middle I (4)	--	1	--	1
Middle II (3)	2	--	--	2
South (2)	1	--	--	1
West (3)	2	1	--	3

All of the written texts and the interviews were analysed through directed content analysis [40]. As a first step, the material, handled as meaning units and descriptive codes, was sorted chronologically according to the implementation process—starting with the formulation of the policy at a national level and ending in the follow-up data at the local level. In the second step, a number of guiding theoretical concepts were used to group the condensed material into larger categories. Most of the concepts can be found in the influential work of Hill and Hupe [22], and their attempt to analyse different layers and critical steps in policy implementation. The categories that were found relevant were summarised in short paragraphs, and illustrative quotes were selected. The process of selecting concepts and grouping categories was dialogically examined by the two researchers responsible for the analysis, until agreement was reached [41]. Examples of guiding concepts and empirical categories are presented in the result section as a summary of results. The study was performed in accordance with Swedish research ethics laws and the Declaration of Helsinki on research ethics. The study was based on informed consent, and all participants were given oral and written information about the aims and the design of the study and their right to withdraw from the study at any time.

## 3. Results and Discussion

### 3.1. The National Strategy

How do national actors handle the issue of including a model for integrated care in a system that is characterised by the division of responsibility between authorities? Looking at the proposal from the National Coordination of Psychiatry, the lack of precision when it comes to describing the proposed model (ACT) was apparent. Common formulations of the “object” for the proposal differed between “the ACT model with case managers” and “a case-management method with ACT orientation”. The text did not provide guidance concerning if the proposal covered all components of the ACT model or just components that were related to the case-manager function. The proposal also did not state if a multi-professional team was a necessary ingredient in the Swedish initiative—in one sentence, “more or less formalised teams” were advocated, which could “create a counterpart to ACT in Sweden”. At the same time, the authors argued for the importance of an implementation with high fidelity in relation to “the model”. The question was, of course, which model and which fidelity scale they were referring to. The promoted initiative was finally supposed to give “skills to deliver treatment, rehabilitation and coordination according to a case management model, especially ACT”.

In the subsequent decision of the government, the text was characterised by a contradictory position that focused on more general CM approaches even though all arguments about evidence for the initiative was derived from research on ACT. Emphasis was put on the case manager functions, not on the team or the organisational principals for the services—in other words, the decision took its starting point in one component of ACT instead of the whole model as a package. In other parts of the document, the decision was formulated in a way that was difficult to interpret. On the one hand, the government wrote that changes toward “case management according to ACT” were important and would require extensive efforts and a redesign of parts of the welfare system. On the other hand, there were formulations saying that integrated services delivered by joint teams were unrealistic. One interpretation is that the government, by presenting this “all or some” approach, had hopes that some places would try to go all in and implement ACT but others only would only choose to use some of the ingredients of the model. 

In summary, the documents that were analysed consisted of messages suggesting that the original ACT model was to be modified and “translated” into a Swedish version even before the ACT model had been tested in practice.

The interviews with the national key players nuanced the motives and the view on the ACT and CM. The representative for the National Coordination of Psychiatry stressed that there was a great deal of pragmatism behind the initiative—the Swedish system was in great need of a function that could manage and coordinate the interventions delivered by both psychiatric health service and the social service authorities. The informant did not believe that it would be possible to work in full accordance with ACT without certain modifications. At the very least, it was suggested that the service providers could strive towards “an ACT-like environment”. One idea was to make the two authorities enter into agreements where they could hire joint case managers. This proposal had been preceded by contacts with representatives for a CM model developed in Sweden under the name Integrated Psychiatry (IP), which was influenced by an international movement called the Optimal Treatment Project (OTP). IP consists of some elements that are well established in EBP, but the model as a whole is not included in the Swedish guidelines for psychosocial interventions. The IP model take its starting point from case managers who, together with the client, put together a so-called resource group consisting of important people such as a psychiatrist, other professionals, and the client’s relatives. The support for the client is planned within the group and guided by a working book with instructions, forms, and instruments for assessment and evaluation. The working book covers things such as family interventions, coping with stress, crisis plans, social skill training, psychological treatment strategies, medication, and somatic health. The following quotation illustrates the informant’s view on this IP model:

Integrated psychiatry is not a competing model to ACT, it is in fact a strategy for implementing the government’s decision. I have tried to analyse it, and I believe that the model in important aspects correlates with the ACT cookbook, if I may say so.

The representative for the National Board for Health and Welfare emphasised the lack of collaboration between authorities as the main reason for implementing CM. One suggestion was that perhaps in the long term the two authorities could replace existing functions as caseworkers or counsellors with case managers. The informant emphasized the concrete hands-on approach as the strength of CM and referred to international research evidence to support the effectiveness of CM. She said that the initiative was focused on CM, not ACT, but she could not describe the connection between the two phenomena:

*Case Management is a method based on ACT, you might say, or perhaps you can express it in the opposite way, that ACT is based on Case Management. Or Case Management that includes ACT*.

The informant pointed out the risk of starting an initiative that only supports the work of psychiatry. She was, therefore, anxious to put forward the perspective of the social services, but she stressed at the same time that she did not want to get involved in the practical performance of the project.

In the interview with the project leader at the Swedish university, he argued for the chosen IP model. Instead of emphasising the principle of the multi-professional team, he focused on the resource-group approach where important actors are invited to planning meetings together with the case manager and the client. This group could, according to the informant, compensate for the absence of a professional team and could also be used to support a broader group of clients even in areas other than psychiatric settings, for example, in primary care. In fact, he stressed the importance of marks against the traditional ACT model:

ACT might be suitable in an Anglo-Saxon context, but here with our model with different authorities, we need to create a kind of Swedish version.

None of the interviewed actors advocated a full implementation of the ACT. Instead, there was a clear “drift” from the evidence-based model. Already at this planning stage there was an adaptation to the Swedish welfare system built into the whole initiative. At the same time, the involved actors picked up arguments for the initiative from research on ACT or intensive CM models—despite the fact that ACT as a concept seemed to disappear in the interviews. One reason for the planned modifications was the fear that an attempt to implement the full ACT model would be too transformative and too radical. Another reason was that the policy makers had determined that the social services should participate significantly in any initiative, and such involvement did not seem possible in the more clinical manner in which services are delivered through ACT teams. Instead, they promoted IP despite the fact that this model was not even mentioned in the preparatory documents or in the government decision. It is clear, in accordance with Hill and Hupe [20], that choices made during the formulation and design of policies have great impact on the implementation process. In this case the drift went in the direction from a well-established program, designed for a limited target group, to a program with a weaker base of evidence that is intended for a broad target group. Thus even before the start of the training program, the problem, the intervention, and the target group had been changed.

### 3.2. Local Experiences of the Training Program

In this section, some of the results from both the interviews with key informants (including follow-up interviews) and the in-depth study will be presented. The participants experienced the programs as ambitious but rather unstructured, in particular the large number of different “working books” was found to be confusing. A common experience was also that the programs were too focused on “psychiatric” matters:

*The* (*short*) *program is very psychiatry-oriented, which is obvious to us working in the social services. I don’t know if that’s in accordance with the intentions, but that’s a fact anyway. The critique among the participants was consistent—very psychiatry-oriented. It was difficult for us to recognise our own role and position in the written education material.*

(Social services participant, municipality West I).

At the South site in the in-depth study, the participants had demanded that the organiser design the program according to the municipality’s local conditions and to the mission of the social service organisation. The initiative resulted in more lecturers with experiences from social work. Similar actions were also taken in the West I and West II municipalities. A common opinion among both managers and staff from the social services was that they did not intend to use the whole IP program that was presented. Instead, they argued for the use of only select parts. Only a few informants stated that they had heard something about ACT in the courses.

The short training program for teams (five weeks) was accomplished at five places spread over as many regions. Each group consisted of about 30–35 participants from both psychiatry and social services. In total, the program reached approximately 150 participants in this first round. The participating staff from the North municipality described the program as very basic and as having the character of information transfer only, but in South and West I and II the orientation was described as practical and having a focus on IP as a concrete strategy.

The long program for case managers (20 weeks) also took place at five locations and included about 15–20 participants at each site for a total of about 100 people. The 20 weeks were distributed over three semesters. The program contained lectures and seminars with national experts covering a number of themes, supervision of client work (in groups of four participants), and training linked to the modules of the IP model. A common opinion was that the content of this program was also poorly adapted to the conditions in the social service organisation:

Everything was presented from a psychiatry perspective, nothing from a social service point of view. It was very good and interesting, but as I said, very psychiatric, and you lost some of the connections with the IP modules and everyday practice.

(Social service participant, South municipality).

Overall, the participants were satisfied with the training-oriented elements in the program. Each participant was supposed to build up and manage a resource group for two of their own clients or patients (so-called “education clients”), and they received continuous supervision from a practitioner experienced in using IP. However, some participants expressed that there was not enough time given to practice all of the modules in the IP model:

I felt that now it is the end of the course, and I haven’t been able to work through all the modules with my clients yet. So what happens now? It became a fuzzy end of the program.

(Social service participant, West II municipality).

In summary, the case of the training programs also seems to be indicative of a soft implementation strategy. The local participating organisations had a large degree of autonomy, and local adaptation of the programs could be negotiated with the project organiser. Despite local variations, emphasis in both training programs was on the IP model and the resource group as a significant instrument in the client work. Sustainable implementation was further complicated by the confusion regarding post-program supervision—the commission included, as mentioned previously, so-called “implementation support” from the program organiser, but whether this really covered supervision in client work was not clearly stated.

### 3.3. The Local Implementation of the Model

In this section, some of the experiences regarding local implementation will be examined. One question is, of course what it was that the local providers were trying to establish—was it a model like ACT, IP, or another version of CM, or was it just limited parts of a model? As was shown in the previous section, the ACT concept was no longer obvious in the program that was being implemented. It was already diluted at the policy level and was not seen as relevant at the local level. Instead, most of the key persons at the regional level that were interviewed, pointed at the CM function as the main purpose of the national initiative. They viewed the mission as local attempts to create a new working role in which a select number of staff would become case managers who would coordinate services for the clients with help from resource groups.

A question to ask, therefore, is how the CM strategies were interpreted. In all 15 municipalities that took part in the programs, at least some attempts were made to prepare space in the organisation for the new concept and for letting the staff that had undergone the training use their new competence in their work, at least for part of the time. There were a few examples where the local actors tried to put together loose-coupled teams with case managers from both psychiatry and social services, but at no site did such arrangements result in integrated teams. In addition, there were no formal demands or instructions in the agreement between the government and the local providers on how the local organisation should be formed during the implementation stage. The university and the private company that was responsible for delivering the training programmes played active roles during the planning and performance of the education, but they pulled back soon after the training programmes were finished. The interviews indicated an uncertainty among a majority of the municipalities regarding the future support from the organiser and the strategies for implementation. One of the key persons in the South region described an attitude on how to deal with the mediated IP model that was recurrent among many informants:

Informant: During the process we have noticed that the model is not suitable for everyone. We can’t help all of our clients. For some, it is not realistic to use all the modules. But you can pick up small parts from the model, or be influenced by the general approach. You can’t be so adherent to the model, just use the parts that you like.

Interviewer: Do you think that the organiser of the training program would like that approach?

Informant: Absolutely not!

The most significant part of the training programmes that remained at the implementation stage was the idea of the resource group. This idea, that the case manager together with the client puts together a group of important people to use for planning and support, was highlighted at almost all sites in the study. Participants in the long programme stated that they had built better relations with both clients and relatives through the use of the resource group. Some of them also described an increased sense of safety and self-esteem among clients as a result of having a functioning resource group.

The IP model consists, as mentioned, of a number of modules that are collected in workbooks. In the follow-up interviews, representatives of the psychiatry services perceived them as useful and worth following. The representatives of the social services, however, expressed doubts about their usefulness because the responsibility of social services does not cover psychiatric services. The part of the IP model that was considered as relevant, besides the CM function and the resource group, was the module about individual plans and crises plans.

The status of CM and IP after the training programmes varied in the local settings. As has been noted, the position of the models was generally stronger in the psychiatry organisations due to the better match between the models and the work carried out by these organisations. A factor of importance seemed to be the access to key persons or purveyors with a large amount of discretion to promote the model as well as having had earlier experiences with similar working methods. In such cases, the strategies with case managers and resource groups could work as platforms for collaboration between the psychiatry and social services.

The sites where the CM function had not even been partially implemented in everyday practice at the time of the follow-up were characterised by a lack of managers who promoted the issue. In these settings, the staff that had been trained as case managers did not receive any formal space in their employment contracts and soon became isolated as “carriers” of the IP concept. The lack of enthusiasm could also be combined with scarce financial resources. This kind of situation was most clearly illustrated in the North municipality in the in-depth study where the lack of interest from politicians and managers was striking:

I don’t know which kind of blessing my boss received from the politicians, but it was never clarified. We tried to invite politicians, managers and civil servants to an information meeting about the national initiative and the training programmes. Nobody came. Not even my boss, so there we sat by ourselves. I have since contacted them for information and updates, but they are not interested.

(Local coordinator, North municipality).

As illustrated earlier, the original ACT model had drifted away in the direction of the IP model, which in turn decomposed into many local versions where specific units from the model were selected. One explanation for the lack of program fidelity at the local level seemed to be the mismatch between the characteristics of the social service authorities and the psychiatric components of the IP model. Another explanation is, of course, the lack of clear governmental steering. In this case, the local providers were given a significant degree of autonomy in both planning and designing their own implementation process, and they were in some respects “left alone” without guidance or support after the training programmes had ended. At six months after the last training program had ended, there were about 100 case managers throughout Sweden working with different mandates, in different organisational settings, with different client groups, and with different parts of the IP model. The single exception seemed to be the strategy of arranging resource groups in collaboration with the clients, although these groups still varied in terms of responsibility and intensity among the case managers. In Table 5 below, the results in terms of empirical categories and descriptions are summarized in accordance to the steps described by Hill and Hupe [20], as they were used as guiding concepts in the analysis.

## 4. Conclusions

The development of community mental health services in the Western world is closely linked to the EBP movement, which is a commonly used strategy to guide politicians, professionals, and local service providers through national guidelines or other knowledge-steering instruments.

**Table 5:** Summary of results.

Concepts	Categories: Descriptions
*Characteristics of the policy*	*Ambiguity*: The policymakers did not seem to be familiar with ACT, and described a somewhat different practice that they sought to implement. *Collaboration*: Collaboration between psychiatry and social services was seen as an end in itself, not as a strategy for reaching high fidelity ACT.
*Formation of policy*	*Single strategy*: The government chose to support the delivery of an education and training program as the only strategy for implementing ACT. *National adaption*: The government involved social services as a core actor, in spite of the clinical psychiatric profile of the training program.
*Steering strategies*	*Soft governance*: The government used stimulation grants and education as steering instruments, without clear demands in relation to the local service providers. *Concept shift*: The providers of the training program were given freedom to design it themselves, and chose to focus on the IP model, rather than ACT. They also chose to focus on a broader group of clients than those usually considered for ACT.
*Response among local agencies*	*Negotiations:* The social services negotiated with the provider of the training program to redesign the education to better fit their organisational conditions, at a price of reducing program fidelity. *Local adaption*: After the training program, most of the participating social service agencies chose to not use the majority of components in the IP-model.
*Inter-organisational relationships*	*Different domains*: Psychiatry and social services have different formal responsibility and were therefore not able to create integrated services or teams. *Compromise*: The idea of “resource groups” was seen as an attractive component—indicating collaboration without building integrated teams.
*Responses from the target group*	*User friendly*: The CM-function was experienced by involved actors as legitimate, through its person centred and flexible approach

There is without question, however, a complex set of challenges involved in the process of transferring the research evidence to local practice. The case of the implementation of ACT in the Swedish context have been used to identify some critical situations in the policy implementation process that have a significant influence on the long-term establishment of evidence-based services. The intention with the design of the study was to collect data from different sources and levels to achieve a comprehensive picture of the project’s impact on community mental health services. This approach means that a wide selection of data was available for this article, but the focus has been on a few actors who are rich in information rather than shallow information from a large number of participants. A risk with a strategy like this is, of course, that important illustrations will be lost—for example, no interviews were made with representatives of the private company that arranged the training programmes or the teachers that were contracted to run them. Instead focus was put on the written material that was connected to the programs. Another weakness in the study is the limited time for follow-up. The researchers only had access to experiences from about one year after the short program and a few months after the long training program were finished. Consequently, the study can say something about the process, the chosen strategies, and the conditions for a sustainable implementation, but it cannot say anything about the actual long-term effects at the local level. In this article, a selection of results that corresponds to the implementation issue will be presented.

Perhaps the simplest conclusion to be drawn from this work is the importance of consider actions on different levels and among different actors to understand the scenarios in which such research-to-practice transitions occur. To better contextualise these challenges, it can be useful to turn back to the steps described by Hill and Hupe [20], and which are further concretised by Johansson [18].

The characteristics of the policy are of importance. In this case, the ideas were based on a solid problem assessment that had identified the need to strengthen collaborations in the provision of mental health services to individual clients. However, the solution was not clearly described and was, in fact, based on misunderstandings about the evidence base. Even as early as the planning stages, the policy-makers had already abdicated from the chance to implement ACT. In an international perspective, it might be reasonable to adjust models based on earlier experiences, as long as attention is paid to the critical ingredients of the current intervention. In this case, however, the “Swedish version” appeared before the “original model” was even tried.

The formation of the policy really seems to affect the implementation process, especially if it concerns state-financed initiatives. The design has to be comprehensive and based on knowledge regarding the most important implementation factors. The example illustrates a single-step strategy where training programs were introduced without complementary strategies for long-term supervision and support. In addition, the political climate seems to have forced the designers to include the social service organisation in the initiative despite the apparent mismatch between the scope of the social service authorities and the chosen model.

The vertical process can appear in many forms depending on the contract between the central and local levels—in Sweden the autonomy of the local municipalities is stated in the constitution, and this affects the ability for governmental actors to govern with “hard” instruments. In this case, the government used exclusively soft steering strategies to implement their policy product, provide stimulation grants, and initiate education programs. No demands were stated in the form of local participation or fidelity to the program. Internationally, the EBP movement is naturally linked to soft governance strategies, especially knowledge steering and information, but there are examples where they can be combined with demands for service providers to act in a certain way to get access to financial support or to avoid negative consequences. The best balance between high program fidelity and reasonable local adaptation seems to be a sensible combination of hard and soft governance.

The responses among local agencies are crucial and can be seen as the final translation of the policy product. The output that reaches the client is shaped by local administrative routines and cultures, and, of course, by the behaviour of the managers and, in the end, the caseworkers. This study has found that the local administrative authorities in Sweden have made extensive adaptations to the IP program—only a few modules persisted during the real contact between staff and clients. On the other hand, these modules seemed to communicate the core values among the participating organisations and indicated a true need to create a collaborative setting around the client. In the absence of detailed steering guidelines and top-down demands, the practice could be highly influenced by strong purveyors or local managers who are actively engaged in the implementation process. This step in the implementation process is, regardless of current routines and instructions, affected by the expertise of the community mental health professionals. A large body of competence, experience, and integrity among managers and staff can support an approach where policies, methods, and research evidence can be handled in a responsible and well-balanced way. The challenge for community mental health services globally is that the field is still only weakly established and consists of a large number of staff with limited experience and short education backgrounds.

Another aspect of the implementation process described by Hill and Hupe is the horizontal inter-organisational relationships between agencies. Sweden can be seen as a good example of this where the distance between different actors limits the ability to implement a policy or, in this case, a model for integrated treatment and support. The formal division of responsibility between social services and psychiatry did, in fact, hamper the ambition of introducing ACT. At the local level, the contacts between the agencies had an impact on both the real-world implementation of the training programs and the more long-term collaboration around the target group simply because of the fact that community mental health services assume a combination of social and psychiatric support. In fact, many of the psychosocial programs that have the most solid evidence base are characterised by their collaborative and integrative approaches.

Finally, the implementation process will be affected by how the clients or the representatives of the target group perceive the policy or the offered services. In general, many of the techniques and approaches associated with community mental health are seen as “user-friendly”, and concepts like the resource group described above are also considered to be popular among users in the local context. This was, of course, a factor that facilitated the promotion of a case manager function within mental health care services in the first place.

Regardless of the general relevance of the chosen case for a discussion about EBP implementation, it is still important to understand how the implementation was experienced. The drift of the concept of ACT was both intended and desired by the national actors. The established EBP model was stepwise translated into a broader coordination function that could be offered to a broader group of users. In practice, this meant that a number of staff in psychiatry as well as in social services were authorized to act as case managers and to put together resource groups. The process also showed how easy it is to rhetorically use the EBP concept to promote and justify initiatives at both the central and local levels. The drift had already begun at the policy development stage and continued during implementation at the regional level where only select parts of the model were used. In addition, the practice between different local settings differed substantially. In the end there is not one consistent model, but instead there is a large number of local versions that will be nearly impossible to scientifically evaluate. It seems that the only general conclusion that can be drawn is that the program consists of about 100 staff around Sweden who have undergone the training program and are providing mental health services in some form to about 300–400 clients. It is easy to argue that strategies for implementing or testing evidence-based models should be designed and governed in a way that allows for systematic evaluation. One basic condition for such evaluations is that the services delivered do not vary too much or that the variation can at least be described in a systematic manner.

The purpose of this paper has been to use a national example to shed light on some important aspects of implementation of evidence-based interventions in community mental health care. Much of the emphasis in implementation research is currently on factors that are team or organisation specific, for example, local needs assessments, recruitment of staff, training, and technical support—factors that are manageable or possible to handle in the local context. These factors were also visible in the current study. However, to analyse elements that influence the implementation process, there is a need to include more levels and actors to really understand the translations that always seem to take place. What happens on one level will in some way affect other levels. An awareness of these simple conclusions is important among both local and central actors. This can help them avoid naive positions when designing and managing such things as national guidelines or when importing internationally evaluated programs. At the very least, such awareness will facilitate an understanding of each actor’s own domains and responsibilities.
